# Microgravity inhibits decidualization via decreasing Akt activity and FOXO3a expression in human endometrial stromal cells

**DOI:** 10.1038/s41598-019-48580-9

**Published:** 2019-08-20

**Authors:** Hye-Jeong Cho, Mi-Ock Baek, Sana Abdul Khaliq, Seung Joo Chon, Kuk Hui Son, Sung Ho Lee, Mee-Sup Yoon

**Affiliations:** 1 Department of Molecular Medicine, School of Medicine, Gachon University, Incheon, 21999 Republic of Korea; 2 Lee Gil Ya Cancer and Diabetes Institute, Gachon University, Incheon, 21999 Republic of Korea; 3 Department of Health Sciences and Technology, GAIHST, Gachon University, Incheon, 21999 Republic of Korea; 4 Department of Obstetrics and Gynecology, Gachon University Gil Medical Center, College of Medicine, Gachon University, Incheon, 21565 Republic of Korea; 5 Department of Thoracic and Cardiovascular Surgery, Gachon University Gil Medical Center, College of Medicine, Gachon University, Incheon, 21565 Republic of Korea; 6 Department of Thoracic and Cardiovascular Surgery, Korea University, Seoul, 02841 Republic of Korea

**Keywords:** Cell proliferation, Environmental impact, Differentiation

## Abstract

Decidualization is characterized by the differentiation of endometrial stromal cells (eSCs), which is critical for embryo implantation and maintenance of pregnancy. In the present study, we investigated the possible effect of simulated microgravity (SM) on the process of proliferation and *in vitro* decidualization using primary human eSCs. Exposure to SM for 36 h decreased the proliferation and migration of eSCs significantly, without inducing cell death and changes in cell cycle progression. The phosphorylation of Akt decreased under SM conditions in human eSCs, accompanied by a simultaneous decrease in the level of matrix metalloproteinase (MMP)-2 and FOXO3a. Treatment with Akti, an Akt inhibitor, decreased MMP-2 expression, but not FOXO3a expression. The decreased level of FOXO3a under SM conditions impeded autophagic flux by reducing the levels of autophagy-related genes. In addition, pre-exposure of eSCs to SM significantly inhibited 8-Br-cAMP induced decidualization, whereas restoration of the growth status under SM conditions by removing 8-Br-cAMP remained unchanged. Treatment of human eSCs with SC-79, an Akt activator, restored the reduced migration of eSCs and decidualization under SM conditions. In conclusion, exposure to SM inhibited decidualization in eSCs by decreasing proliferation and migration through Akt/MMP and FOXO3a/autophagic flux.

## Introduction

Human space exploration has been growing in recent years. This has led researchers to investigate the effect of harsh environmental conditions, including extreme temperature, ionizing radiation, and altered gravity. Among them, exposure to microgravity has been reported to result in various detrimental effects on the muscular mass^1^, immune system^2^, cardiovascular system^3^, bone mass^1^, nervous system^4^, and endocrine system^5^. Conditions of near weightlessness affects cell growth, differentiation, apoptosis, and autophagy in different cells^6^. However, the effect of microgravity on embryo implantation and maintenance of pregnancy in the human endometrium has not been examined yet.

Decidualization, characterized by the differentiation of endometrial stromal cells, is a profound change in the cells of the endometrium for embryo implantation and maintenance of pregnancy^7^. During the process of decidualization, the fibroblast-like endometrial stromal cells (eSCs) acquire a round shape, with accumulation of glycogen and lipids, secretion of growth factors and cytokines, such as prolactin (PRL) and insulin-like growth factor binding protein 1 (IGFBP1), and accumulation of extracellular matrix (ECM). Although progesterone/cAMP are well known inducers of decidualization, mechanical stretch in the endometrium has been reported to induce decidualization by regulating the expression of IGFBP-1^7^. In addition, mechanical stretch is transduced into biochemical signals via interleukin (IL)-8 to regulate endometrial differentiation^8^. Subendometrial myometrial contraction also has been shown to occur throughout the menstrual cycle, which is related to other uterine movements, such as endometriosis and mentruation^9^. These findings led us to investigate the possible effect of microgravity on the proliferation and differentiation of eSCs.

In the current study, we hypothesize that simulated microgravity (SM) is a critical regulator of the decidualization of human eSCs. To verify this hypothesis, we examined the changes in the proliferation and migration of SM-exposed eSCs and analyzed the effects of decidualization exposure to SM before the initiation of decidualization. Taken together, we suggested that mechanical loading is a critical factor in the regulation of proliferation and decidualization in human eSCs, proposing an airspace strategy to protect our body.

## Results

### SM inhibits the growth of primary human eSCs without cell death

**Figure 1:**
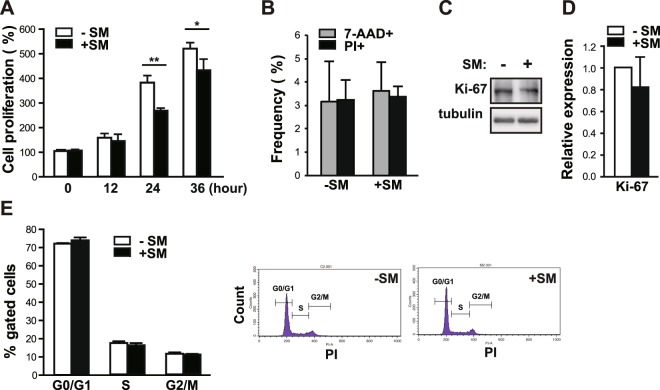
SM inhibits the growth of human eSCs without any changes in cell death and cell cycle progression. (**A**) Primary human eSCs were incubated either under terrestrial gravity (1 *g*) or under SM for indicated time periods and counted using a cell counter. (**B**) The cells were treated as (**A**) for 36 h, stained with either 7-AAD or PI, and then analyzed by flow cytometry. (**C**) The cells were treated as in (**B**), lysed, and analyzed by western blotting. (**D**) Western blot images were analyzed using ImageJ to determine the relative protein expression of Ki-67 (using tubulin as the internal control). (**E**) The cells were treated as (**B**), stained with PI and analyzed using flow cytometry. Abbreviations: simulated microgravity (SM); 7-aminoactinomycin D (7-AAD); propidium iodine (PI). Data are expressed as mean ± standard deviation (SD), with paired *t*-tests performed as indicated. *P < 0.05, **P < 0.01 versus control at each indicated time.

We first examined whether a reduced gravitational force affects the growth rate of primary human eSCs using a clinostat, a device that is widely used for generating SM. Primary eSCs grown to 80–90% confluency were placed in either a dynamic reactor to simulate 0 *g* or in a stationary control (1 *g*). After rotation of the reactor in both vertical and horizontal planes, the number of eSCs was measured at 0, 12, 24, and 36 h. Human eSCs grew at a significantly slower rate under SM conditions (30 and 17% reduction was seen at 24 and 36 h, respectively) compared to those grown under terrestrial gravity (Fig. 1A). The percentage of dead cells under SM conditions remained unchanged compared to those under conditions of terrestrial gravity, as indicated by the number of both 7-aminoactinomycin D (7-AAD)^+^ and propidium iodine (PI)^+^ cells (Fig. 1B), suggesting no difference in viability between either condition. Consistent with these findings, the level of Ki-67 in SM-exposed eSCs was mildly decreased, but not significantly, compared to that of a stationary control (Fig. 1C,D). As shown in Fig. 1E, the ratios of cells in the G1, G2, and S phases under SM conditions were comparable to those of cells under terrestrial gravity, indicating no changes in the progression of cell cycle under SM conditions. These results indicate that exposure to SM reduces the proliferation of eSCs without a discernible increase in the apoptotic cell fraction or changes in cell cycle progression.

### SM inhibits the migration of primary human eSCs

**Figure 2:**
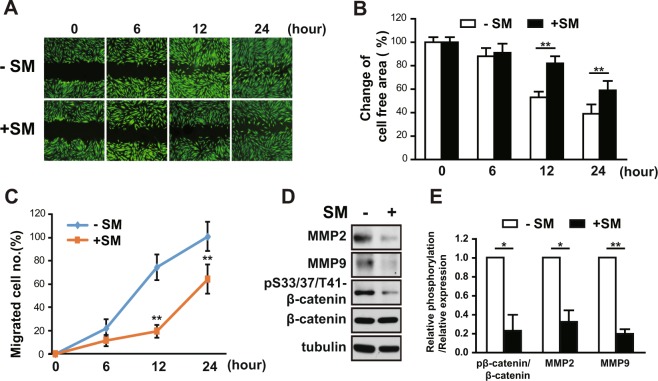
SM impedes the migration of human eSCs. (**A**–**C**) Human eSCs were scratched with a T200 tip and then incubated under 1 *g* or SM for the indicated times. (**A**) Cells were stained using the CytoPainter Cell Tracking Staining Kit and photographed. (**B**) The cell-free area was measured using ImageJ and change of cell-free area was calculated. (**C**) The number of migrated cells was counted using ImageJ. (**D**) The cells were incubated either under terrestrial gravity (1 *g*) or under SM for 36 h, lysed, and analyzed by western blotting. (**E**) Western blot images were analyzed using ImageJ to determine the relative protein expression of MMP-2 and MMP-9 (using tubulin as the internal control) and the relative phosphorylation of S33/37/T41-β-catenin (using β-catenin as the internal control). Abbreviations: simulated microgravity (SM). Data are expressed as mean ± SD, with paired *t*-tests performed as indicated. **P < 0.01, *P < 0.05 versus control at each indicated time.

Migration of human eSCs is required for human embryonic trophoblast invasion^10^. We analyzed the effect of SM on the migration of primary eSCs using a wound healing scratch assay. After 0, 6, 12, and 24 h under SM conditions, we examined cell motility by assessing changes in both cell-free area and the number of migrated cells. Exposure of eSCs to SM caused a significant decrease in cell motility of the stained image of cells (Fig. 2A), increase in the remaining cell-free area (Fig. 2B), and decrease in the number of migrated cells (Fig. 2C). The remaining cell-free area and number of migrated cells at 12 h after SM exposure were increased by 1.6-fold and decreased by 3.7-fold, respectively, as compared to exposure to 1 *g*, suggesting that eSCs under SM migrate evidently slower (Fig. 2B,C). Consistent with this observation, the level of MMP-2 and MMP-9, well-known regulators that degrade the ECM, and the phosphorylation of β-catenin, a component of cell to cell connection^11^, were decreased in eSCs exposed to SM for 36 h (Fig. 2D,E), thereby confirming that exposure to SM leads to slow migration of eSCs.

### SM reduces Akt activity in human eSCs

**Figure 3:**
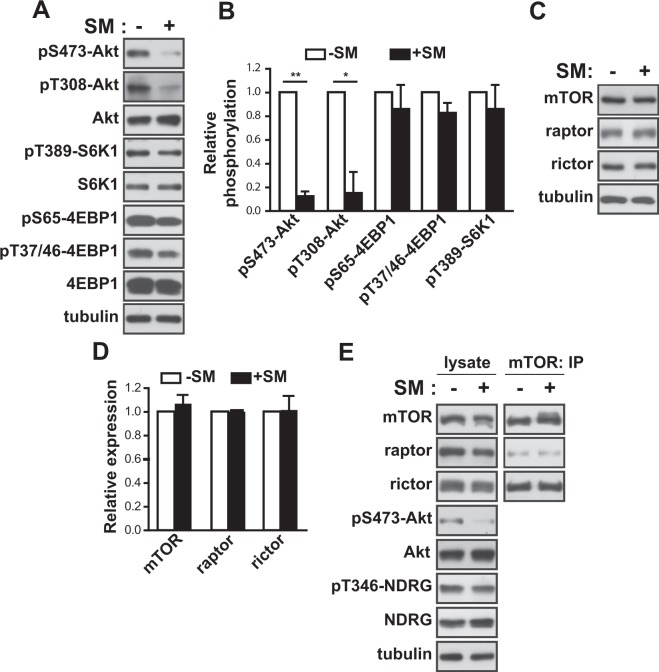
SM decreased Akt activity in human eSCs. (**A**–**D**) Human eSCs were incubated either under terrestrial gravity (1 *g*) or under SM for 36 h, lysed, and analyzed by western blotting (**A,C**). Western blot images were analyzed using ImageJ to determine the phosphorylation of pS473-Akt relative to Akt, pT308-Akt relative to Akt, pT389-S6K1 relative to S6K1, pS65-4EBP1 relative to 4EBP1, and pT47/36-4EBP1 relative to 4EBP1 (**B**), and the expression of mTOR, raptor, and rictor relative to tubulin (**D**). The cells were treated as in (A), subjected to immunoprecipitation using antibodies against mTOR, and analyzed by western blotting. Abbreviations: simulated microgravity (SM). Data are expressed as mean ± SD, with paired *t*-tests performed as indicated. *P < 0.05, **P < 0.01 versus control at each indicated time.

**Figure 4:**
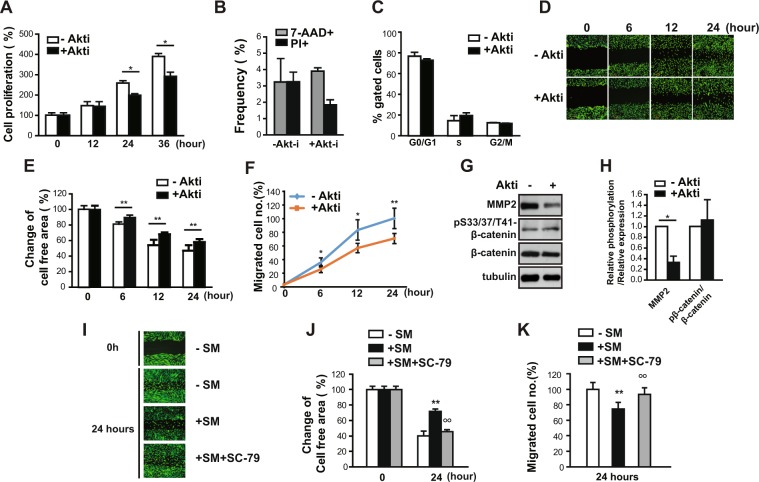
Akt decreased cell growth and migration in human eSCs. (**A**) Human eSCs were incubated with or without 1 μM Akti for the indicated times. The cells were counted using a cell counter. (**B**) The cells were treated as in (**A**), stained with either 7-AAD or PI, and analyzed by flow cytometry. (**C**) The cells were treated as in (**A**), stained with PI, and analyzed by flow cytometry. (**D**–**F**) The cells were scratched with a T200 tip and then incubated with or without 1 μM Akti for the indicated times. (**D**) The cells were stained using the CytoPainter Cell Tracking Staining Kit and photographed. (**E**) The cell-free area was measured using ImageJ and change of cell-free area was calculated. (**F**) The number of migrated cells was counted using ImageJ. (**G,H**) The cells were treated as in (**A**), lysed, and subjected to western blotting. (**H**) Western blot images were analyzed using ImageJ to determine the phosphorylation of pS33/37/T41-β-catenin relative to β-catenin and the expression of MMP-2 relative to tubulin. (**I**–**K**) The cells were scratched with a T200 tip and incubated with or without 0.2 μg/ml SC-79 under SM conditions for 24 h. (**I**) The cells were stained using the CytoPainter Cell Tracking Staining kit and photographed. (**J**) Cell-free areas were measured using ImageJ and the changes in the cell-free areas were calculated. (**K**) The number of migrated cells was counted using ImageJ. Abbreviations: simulated microgravity (SM); 7-aminoactinomycin D (7-AAD); propidium iodine (PI). Data are expressed as mean ± SD, with paired *t*-tests performed as indicated. *P < 0.05, **P < 0.01 versus control at each indicated time; °°*P* < 0.01 versus SM exposed cells.

Akt is critical to the regulation of cell migration and growth^12^. Next, we examined the phosphorylation of Akt to assess the activity of Akt. Akt phosphorylation at both serine 473 and threonine 308 decreased significantly (up to 88 and 85%, respectively) in eSCs exposed to SM for 36 h (Fig. 3A) compared to that in cells under 1 *g* (Fig. 3B). Akt promotes cell growth via translational regulation by activating the mTOR complex1 (mTORC1)^13^. However, the phosphorylation of S6K1 (at threonine 389) and eukaryotic initiation factor 4E binding protein 1 (4EBP1) (at serine 65 and at threonine 37 and 46), which are well known downstream targets of mTORC1, was not changed significantly (Fig. 3A,B). Exposure to SM for 36 h did not affect the expression of mTOR, raptor, and rictor, which are the major components of mTORC1 and mTORC2 (Fig. 3C,D). In addition, the level of either raptor or rictor in the mTOR complexes remained unchanged (Fig. 3E). Interestingly, the phosphorylation of NDRG, a downstream target of SGK that is regulated by mTORC2, also remained unchanged under SM conditions (Fig. 3E), indicating that the decrease in Akt phosphorylation did not originate from the inhibition of mTORC2 activity. Next, to examine the involvement of Akt in the growth and migration of cells, we treated eSCs with Akti, a selective inhibitor of Akt, for the indicated time periods. The growth of eSCs decreased (Fig. 4A) by 23% at 24 h and 25% at 36 h, with no significant changes in cell death and cell cycle progression, as evidenced by the frequencies of 7-AAD^+^ and PI^+ ^cells (Fig. 4B) and the ratios of cells in the G1, G2, and S phases (Fig. 4C), respectively. In addition, the migration of eSCs was reduced in the presence of Akti, as shown by the stained images of eSCs with reduced motility (Fig. 4D), the increased free area between cells (Fig. 4E), and the deceased number of migrated cells (Fig. 4F). Treatment with Akti significantly reduced the expression level of MMP-2. However, the phosphorylation of β-catenin remained unchanged in the presence of Akti (Fig. 4G,H), indicating that reduction of β-catenin phosphorylation might be involved in the decrease of cell motility in an Akt-independent manner. Insulin induced the phosphorylation of Akt, but not β-catenin in human eSCs (SFig. 1), supporting the Akt-independent regulation of β-catenin phosphorylation. Moreover, treatment with SC-79, an Akt activator, restored the reduced migration of human eSCs under SM conditions (Fig. 4I,J,K), confirming that Akt regulates the migration of eSCs under SM conditions. These results suggested that exposure to SM inhibited the growth and migration of eSCs through inactivation of Akt, resulting in a decrease of MMP-2 expression.

### SM suppresses FOXO3a protein expression

**Figure 5:**
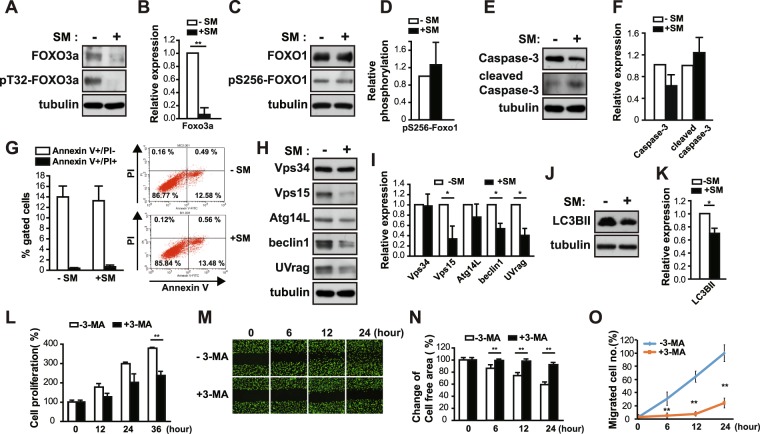
FOXO3a expression and autophagic flux decreased under SM condition in human eSCs. (**A**–**F,H**–**K**) Human eSCs were incubated either under terrestrial gravity (1 *g*) or under SM for 36 h, lysed, and subjected to western blotting. (**B,D,F,I,K**) ImageJ was used to analyze the following: the expression level of FOXO3a relative to tubulin (**B**), the phosphorylation of pS256-FOXO1 relative to FOXO1 (**D**), the expression level of caspase-3 and cleaved caspase-3 relative to tubulin (**F**), the expression level of Vps34, Vps15, Atg14L, beclin1, and UVrag relative to tubulin (**I**), and the expression of LC3BII relative to tubulin (**K**). (**G**) Cells were treated as in (A), stained with PI and annexin V, and analyzed by flow cytometry. (**L**) Cells were incubated with or without 10 mM 3-MA for the indicated times and counted using cell counter. (**M**–**O**) The cells were scratched with a T200 tip and then treated as in (**L**) for the indicated times. (**M**) Cells were stained using the CytoPainter Cell Tracking Staining Kit and photographed. (**N**) The cell-free area was measured using ImageJ and change of cell-free area was calculated. (**O**) The number of migrated cells was counted using ImageJ. Abbreviations: simulated microgravity (SM); propidium iodine (PI); 3-methyladenine (3-MA). Data are expressed as mean ± SD, with paired *t*-tests performed as indicated. *P < 0.05, **P < 0.01 versus control at each indicated time.

**Figure 6:**
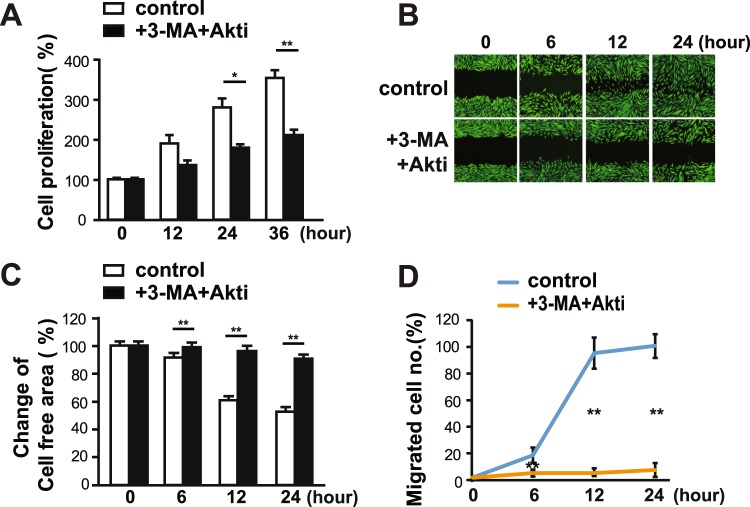
Both Akt and autophagic flux regulated cell migration under SM condition in human eSCs. (**A**) The cells were incubated with or without both 10 mM 3-MA and 1 μM Akti for the indicated times and counted using a cell counter. (**B**–**D**) The cells were scratched with a T200 tip and treated as in (**A**) for the indicated times. (**B**) The cells were stained using the CytoPainter Cell Tracking Staining kit and photographed. (**C**) The cell-free areas were measured using ImageJ and changes in cell-free areas were calculated. (**D**) The number of migrated cells was counted using ImageJ. Abbreviations: simulated microgravity (SM); 3-methyladenine (3-MA). Data are expressed as mean ± SD, with paired *t*-tests performed as indicated. *P < 0.05, **P < 0.01 versus control at each indicated time.

FOXO3a regulates the transcription of MMPs in decidualized human eSCs^14^. In order to investigate the involvement of FOXO3a in SM-induced migration through control of MMP-2 expression, we next examined the expression and phosphorylation of FOXO3a under SM conditions. Akt phosphorylates FOXOs to inhibit their transcriptional activity by exporting FOXOs from the nucleus^15^. The level and phosphorylation of FOXO3a decreased in eSCs exposed to SM for 36 h (Fig. 5A,B). The phosphorylation and level of FOXO1 remained unchanged (Fig. 5C,D), suggesting that decrease in FOXO3a expression is not a common phenotype in FOXOs under SM conditions. FOXO3a is involved in the transcriptional regulation of proteins in diverse cellular pathways, including cell cycle inhibition, autophagy, and apoptosis^15^. No significant reduction in the level of caspase-3 and cleaved caspase-3, a central regulator of apoptosis, was observed (Fig. 5E,F), and the population of apoptotic cells remained unchanged in eSCs under SM conditions compared to that in cells under 1 *g* condition, as shown by fluorescence-activated cell sorting (FACS) analysis using annexin-V/propidium iodine (PI) double staining (Fig. 5G). Exposure of eSCs to SM decreased the expression of autophagy-related regulators, including Vps15, beclin1, and UVrag (Fig. 5H,I). The level of LC3BII, the representative marker of autophagic flux, decreased, indicating a decrease in autophagic flux (Fig. 5J,K), which agreed with the decrease in autophagic gene expression. The level of p62 protein decreased in eSCs under SM conditions (SFig. 2B,C), due to a reduction in p62 mRNA expression (SFig. 2A). Consistent with these results, treatment with 3-methyladenine (3-MA), an inhibitor of autophagy that inhibits Vps34, decreased the growth of eSCs (Fig. 5L) as well as the migration of eSCs, as indicated by the images of migrated cells (Fig. 5M), change of cell-free area (Fig. 5N), and number of migrated cells (Fig. 5O). In addition, co-treatment of eSCs with 3-MA and Akti resulted in the highly reduced proliferation (Fig. 6A) and migration of eSCs (Fig. 6B,C,D). These results indicate that a decrease in Akt activity and autophagic flux may induce slow migration under SM conditions. However, FOXO3a level remained unchanged after treatment with Akti, although FOXO3a phosphorylation decreased (SFig. 3), suggesting that FOXO3a expression level under SM conditions is regulated in an Akt-independent manner.

### SM suppresses the decidualization of primary human eSCs

**Figure 7:**
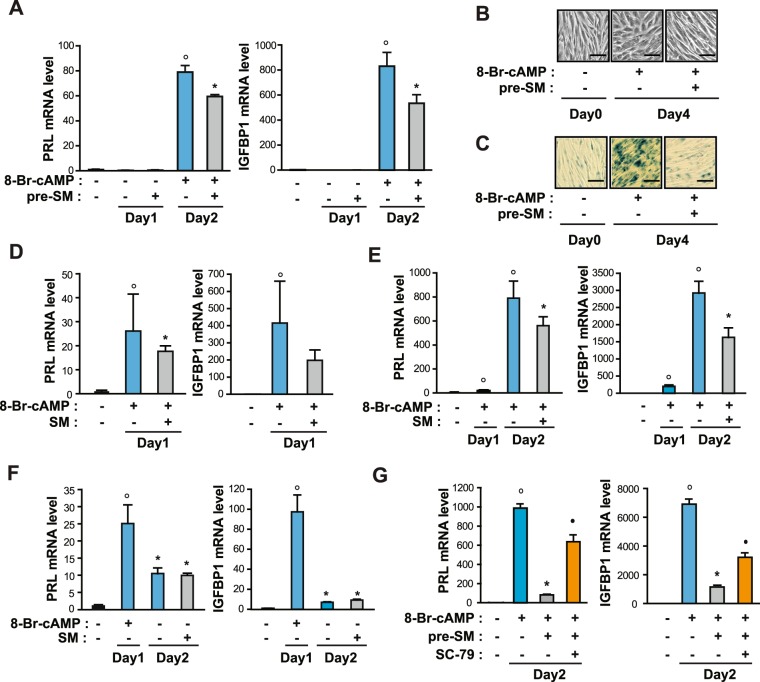
SM decreased decidualization of human eSCs. (**A**) Human eSCs were incubated either under 1 *g* or SM for 1 day, after which they were induced to differentiate in the presence of 0.5 mM 8-Br-cAMP under 1 *g* for 1 day. Cells were lysed and subjected to a quantitative real time PCR (qRT-PCR) analysis. (**B**) The cells were treated as in (**A**), induced to decidualization for 4 days, (**C**) stained using senescence associated β-galactosidase staining kit, and photographed under microscope. Scale bar = 50 μm. (**D**) The cells were treated with 8-Br-cAMP under 1 *g* or SM for 1 day, lysed, and analyzed by qRT-PCR. (**E**) The cells were differentiated in the presence of 8-Br-cAMP for one day and shifted to either 1 *g* or SM for one day in the presence 8-Br-cAMP. The cells were then subjected to qRT-PCR. (**F**) The cells were differentiated as in (**D**) and shifted to either 1 *g* or SM for one day in the absence of 8-Br-cAMP. (**G**) The cells were incubated under SM with or without 5 μg/ml SC-79 for 1 day, after which they were induced to differentiate in the presence of 8-Br-cAMP under 1 *g* for 1 day. The cells were lysed and subjected to a qRT-PCR analysis. Abbreviations: simulated microgravity (SM); simulated microgravity for 24 h before the induction of differentiation (Pre-SM); prolactin (PRL); insulin-like growth factor binding protein 1 (IGFBP1). Data are expressed as mean ± SD, with paired *t*-tests performed as indicated. °*P* < 0.05 versus undifferentiated control; *P < 0.05 versus differentiated control without Pre-SM; ^•^*P* < 0.05 versus differentiated cells with Pre-SM.

Akt and FOXO3a are critical regulators of decidualization of eSCs^14^. Next, we proceeded to test whether SM affects the efficiency of decidualization in eSCs. First, we pre-exposed the cells to either SM or 1 *g* conditions for one day and then induced decidualization by shifting to differentiation medium with 0.5 mM 8-Br-cAMP for one day. Pre-exposure to SM significantly suppressed decidualization, which was indicated by a decrease in the mRNA expression of PRL and IGFBP1 (Fig. 7A), decidua-like morphological changes (Fig. 7B), and senescence-associated β-galactosidase (SAβG)^+^ cells, which were reported to increase during decidualization^16^ (Fig. 7C), suggesting that pretreatment with SM results in defective decidualization. When eSCs were induced for decidualization under SM conditions for one day, the mRNA levels of PRL and IGFBP1 were reduced (Fig. 7D). In addition, exposure to SM for one day after the induction of decidualization by adding 8-Br-cAMP inhibited the mRNA expression of PRL and IGFBP1 (Fig. 7E). However, when decidualized human eSCs were restored to an undifferentiated phenotype upon withdrawal of 8-Br-cAMP, as previously reported^17^, the reverse process of decidualization under SM condition was comparable to that under 1 *g* condition (Fig. 7F), indicating no effect of SM exposure on restoration of growth status in human eSCs. When the eSCs were pre-exposed to SM in the presence of SC-79 for one day and induced to differentiation by the addition 8-Br-cAMP, the mRNA expression of PRL and IGFBP1 was partially restored (Fig. 7G), suggesting that the inhibition of Akt under SM conditions resulted in decidualization defects. These results suggested that exposure to SM reduced decidualization specifically in eSCs.

## Discussion

**Figure 8:**
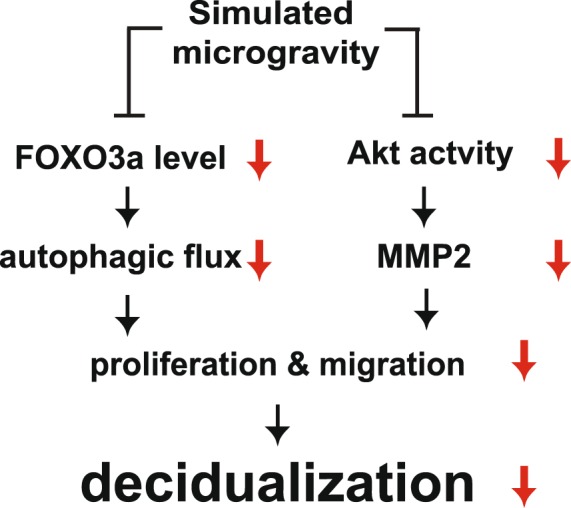
The proposed model of the regulation of decidualization in human eSCs under SM. Exposure of human eSCs to SM decreased FOXO3a expression level and Akt activity, leading to the blockage of autophagic flux and MMP-2 expression, respectively. This reduced the growth and migration of human eSCs, resulting in defective decidualization.

Decidualization is required to facilitate implantation and maintain pregnancy^18^. Subendometrial myometrial movement in the endometrium induces biochemical signals, which trigger endometrial physiological and pathological responses^9^. Although mechanical loading has been shown to promote decidualization^7^, the mechanism by which it controls the differentiation of human eSCs is unclear. Here, we showed that exposure to SM (mechanical unloading by a clinostat) inhibited decidualization in eSCs by modulating their proliferation and migration. Exposure to SM decreased Akt activity and FOXO3a expression, leading to suppression of MMP-2 expression and autophagic flux, respectively. Hence, we propose that mechanical unloading inhibits decidualization through inhibiting Akt- and FOXO3a-dependent cell growth and migration (Fig. 8).

Inhibition of decidualization under SM condition suggested that mechanical unloading plays a role in the differentiation of eSCs. We found that exposure to SM suppressed decidualization during both the initial and middle stages of decidualization (Fig. 7D,E). Mechanical loading enhances decidualization through stimulation of IL-8 secretion^8^, and the production and secretion of IGFBP1^7^. Notably, when the cells were pre-exposed to SM before initiation of decidualization, the expression of the decidualization markers, PRL and IGFBP1, and morphological change were significantly decreased (Fig. 7A,B), indicating that changes in the growth rate of eSCs could induce profound effects on the cells, resulting in defective decidualization. In agreement with our observation, the efficiency of decidualization is determined by both the growth rate and the extent of migration of eSCs^19^.

Akt activity directly and indirectly regulates cell migration by regulating the actin cytoskeleton^20^, cell-cell adhesion, cell motility, and extracellular degradation^12^. Decreased Akt activity induces mesenchymal to epithelial transition (MET), a counterpart of epithelial to mesenchymal transition, which increases cell-cell adhesion and reduces cell motility^21^. Induction of decidualization also inhibits Akt activity, resulting in partial induction of MET-like molecular changes^21^. In the current study, exposure to SM decreased cell migration through regulation of Akt activity (Fig. 4I,J,K), resulting in the inhibition of decidualization during the initial and middle stages of differentiation (Fig. 7). These results suggest that Akt activity is crucial for maintaining the decidualization potential of eSCs. However, decrease in Akt phosphorylation was accompanied by neither phosphorylation of S6K1, 4EBP1 (mTORC1 activity; Fig. 3A), nor NDRG (mTORC2 activity; Fig. 3E). The composition of the mTOR complex was also not affected (Fig. 3E). These results suggested that another regulation pathway may induce the decrease in Akt phosphorylation under SM conditions. Since the regulation of Akt activity by phosphatases, such as PP2A in endometrial cancer^22^ and FKBP51 in the decidualization of human eSCs^23^ has been reported in human eSCs, SM may activate Akt-specific phosphatases, which resulted in a decrease in Akt phosphorylation. Whether PP2A or FKBP51 is involved in SM-induced decrease in Akt activity warrants further investigation.

Remodeling of the extracellular environment is a key event in decidualization. MMPs are responsible for cleaving the ECM components and process ECM-tethered growth factors for tissue remodeling^24^, which is critical for successful decidualization. Activin, a positive regulator of decidualization, promotes the expression of MMPs^25^, whereas TGF-β suppresses endometrial MMP activity^26^. MMP-9 also produces steroid response component 1-isoform C in a TNF-α dependent manner in the endometriotic mouse tissue^27^, suggesting that MMPs regulate endometrial physiology in diverse ways. We found that Akt inhibition induces slow migration by decreasing MMP expression in eSCs, as previously shown in melanoma^28^, breast cancer cells^29^, vascular smooth muscle cells^30^, and human rheumatoid arthritis fibroblast-like synoviocytes^31^. Whether Akt directly regulates MMP expression needs to be further investigated in eSCs.

β-catenin is a transmembrane protein, which is associated with E-cadherin and involved in cell adhesion. β-catenin is also a component of the Wnt signaling pathway^32^. In the absence of a Wnt signal in normal cells, β-catenin forms a complex, which includes glycogen synthase kinase 3β (GSK-3β). GSK-3β phosphorylates β-catenin, targeting it for ubiquitin-dependent degradation by the proteasome, thereby maintaining a low level of free cytoplasmic β-catenin^32^. In the present study, β-catenin phosphorylation in human eSCs decreased in an Akt-independent manner under SM conditions (Fig. 4G,H). A previous report shows that PTEN regulates nuclear localization and pSer675-β-catenin independent of the PI3K–AKT–GSK3b axis^33^. Thus, a novel regulatory signaling of Wnt/ β-catenin independent of PI3K/Akt may exist, which warrants further investigation.

The expression of FOXO has been shown to be differentially regulated in human eSCs^34^. FOXO3a is expressed in undifferentiated eSCs, but not in decidualized eSCs. We found that exposure to SM significantly reduced FOXO3a expression (Fig. 5A), leading to a decrease in autophagic flux (Fig. 5J,K). FOXO3a is a requisite for sustaining autophagy under low nutrient conditions^35^. Basal autophagic flux is dependent on mTOR signaling, and the induction or maintenance of autophagic flux was determined by FOXOs in muscle atrophy^35^. Because of the redundancy of FOXO family members in muscle cells, the deletion of a single member of the FOXO family does not suppress autophagy^35^. However, SM exposure-induced FOXO3a deletion was sufficient to inhibit the expression of autophagic genes and subsequent autophagic flux in undifferentiated eSCs. It is possible that FOXO3a is the main member of the FOXO family that maintains autophagy in undifferentiated eSCs, since FOXO3a is highly expressed in those cells, while FOXO1 is expressed in the decidualized endometrium *in vitro*^36^ and *in vivo*^34^, and FOXO4 is not expressed in the normal endometrium^34^. The role of autophagy in migration has been recently demonstrated^37^. Inhibition of autophagy decreases the rate of cell motility by stabilizing focal adhesions, subsequently resulting in the reduction of migration rate^37^. The association between autophagosome and focal adhesions facilitates the destabilization and turnover of cell-matrix contacts via focal adhesion proteins^38^. Decreased autophagic flux stabilizes cell-matrix contacts under SM conditions in eSCs and simultaneously, a low level of MMP-2 further inhibits cell matrix degradation.

In the present study, we used specialized culture dishes, which are porous to air and completely sealed, in order to maintain the culture system during the rotation of human eSCs on the clinostat. Due to the lack of a suitable culture system for the current clinostat, the current study has limitations, since we were unable to test the effect SM exposure on extracellular biomatrix invasion and the 3D culture of eSCs. As such, this warrants further investigation.

Mechanical unloading by exposure to SM altered cell growth as well as decidualization in eSCs. Our study provides the first evidence that decidualization was restrained under SM conditions via a decrease in Akt activity and FOXO3a expression. The decrease in Akt activity and autophagic flux led to slow cell growth and migration, resulting in low efficiency of decidualization. Taken together, our findings suggest that the microgravity during spaceflight could lead to an unreceptive endometrium by suppressing decidualization potential.

## Methods

### Antibodies and other reagents

Antibodies were obtained as follows: anti-raptor and -rictor antibodies were from Bethyl Laboratories (Montgomery, TX, USA); all other primary antibodies were from Cell Signaling Technology (Danvers, MA, USA). All secondary antibodies were from Jackson ImmunoResearch Laboratories Inc. (West Grove, PA, USA). All other reagents were from Sigma-Aldrich (St. Louis, MO, USA).

### Isolation and culture of human eSCs

Human eSCs were isolated from the human endometrium, which was obtained by hysterectomy from 25 premenopausal women, aged 40–45 years. The participants underwent surgery for non-endometrial abnormalities at Gil Hospital between August 2018 and January 2019. All procedures were approved by Gachon University and the Institutional Review Board (IRB) (Permission number: GAIRB2018–301). All experiments were performed in accordance with the relevant guidelines and regulations. Informed consent was obtained from all participants. Isolation of eSCs was performed following a previously described procedure^17^. Human eSCs were grown in Dulbecco’s modified Eagle’s medium (DMEM) containing 1 g/L glucose with 10% fetal bovine serum (FBS) at 37 °C and 5% CO_2_ and detached from the plate using 0.05% trypsin-EDTA (Welgene, Gyeongsangbuk-do, Korea). To induce *in vitro* decidualization, cells were plated, grown to 100% confluence, treated with DMEM with 10% FBS containing 0.5 mM 8-Br-cAMP, and replenished with fresh medium every other day. The cells were stained with senescence-associated β-galactosidase (senescence-associated β-galactosidase staining kit, Cell Signaling Technology).

### SM: the clinostat system

To induce SM on the ground, a clinostat system (3D clinostat, Shamhantech Inc., Bucheon, Korea) was used in this study. Human eSCs were plated in a membrane cell culture dish (SPLPermea^™^; SPL Life Sciences Co., Gyeonggi-do, Korea). The cells were attached to the cell culture dish, which was filled with culture medium. The dish was fixed carefully to the rotating panel of the clinostat system, which was then placed in an incubator at 37 °C with a 5% CO_2_ atmosphere. The clinostat was continuously rotated at 5 rpm for 36 h. The control cells (normal gravity) were plated on the same type of dish and incubated in the same incubator as the cells exposed to SM but did not undergo clinorotation.

### *In vitro* scratch wound healing assay

Human eSCs were incubated in DMEM containing 1.0 g/L glucose supplemented with 10% FBS. The medium was then replaced with DMEM (0.1% FBS), after which the cells were incubated at 37 °C in an atmosphere of 5% CO_2_ for 18 h to minimize cell proliferation. An artificial wound was created by disrupting the monolayer using a sterile plastic pipette tip (200 µL). A migration assay was then performed in the presence or absence of SM at 6, 12, and 24 h. The cells were then stained using the CytoPainter Cell Tracking Staining Kit (Abcam, Cambridge, MA, USA) following the manufacturer’s protocol. Images were captured using a laser Scanning Microscope 700 (Carl Zeiss, Oberkochen, Germany) equipped with a 5× objective. Cell migration was measured as the percentage of the remaining wound area relative to the cell-free area of the initial scratch. The number of migrated cells was calculated using an ImageJ cell counter. All experiments were performed in at least triplicate.

### Cell lysis, immunoprecipitation, and western blot analysis

Human eSCs were washed once with ice-cold phosphate buffered saline (PBS), scraped and then lysed with lysis buffer (Cell Signaling Technology). The supernatant was collected after microcentrifugation at 13,000 *g* for 10 min, and then boiled in sodium dodecyl sulfate sample buffer for 5 min. Immunoprecipitation was performed with anti-mTOR antibody, followed by incubation with protein G agarose for 1 h at 4 °C. For immunoprecipitation, lysis buffer containing 40 mM 4-(2-hydroxyethyl)-1-piperazineethanesulfonic acid (pH 7.4), 120 mM NaCl, 10 mM pyrophosphate, 50 mM NaF, 10 mM β-glycerophosphate, 2 mM EDTA, 1X Sigma protease inhibitor cocktail, and 0.3% 3-[(3-cholamidopropyl)dimethylammonio]-1-propanesulfonate was used. Western blotting was performed as previously described^36^.

### Quantitative real-time (RT)-PCR

Total RNA was extracted from human eSCs under either terrestrial gravity or SM. Quantitative RT-PCR was performed following a previously described protocol^36^. Human glyceraldehyde 3-phosphate dehydrogenase (GAPDH) was used to normalize gene expression. The following primers were used; *PRL*, Forward: GGAGCAAGCCCAACAGATGAA, Reverse: GGCTCATTCCAGGATCGCAAT; *IGFBP1*, Forward: TTGGGACGCCATCAGTACCTA, Reverse: TTGGCTAAACTCTCTACGACTCT; *GAPDH*, Forward: GGAGCGAGATCCCTCCAAAAT, Reverse: GGCTGTTGTCATACTTCTCATGG.

### Cell proliferation and viability

The number of trypan blue (Welgene, Gyeongsangbuk-do, Korea)-stained cells was counted to assess cell viability, according to the dye exclusion method^39^ using a cell counter (LUNA-II™ Automated Cell Counter, Gyenggi-do, Korea). All counts were performed in duplicate with independent samples after 12, 24, and 36 h of growth. To analyze cell viability, cells were collected by centrifugation at a concentration of 3 × 10^5^ cells/tube, incubated with either 7-AAD (50 μg/mL, Biolegend, San Diego, CA, USA) for 10 min at room temperature, or PI (50 mg/L) and 1.5% of RNase A (7 mg/mL) for 30 min at 37 °C in the dark. The number of 7-AAD or PI-stained cells was counted using flow cytometry analysis (BD FACS Calibur; BD Biosciences, San Jose, CA, USA).

### Analysis of the cell cycle and apoptosis

The cells were collected by centrifugation at a concentration of 3 × 10^5^ cells/tube and washed twice with PBS after exposure to SM for 36 h. The cell pellets were suspended in 1 mL ice-cold 70% ethanol at 4 °C for 1 h and washed with PBS once. The cells were then resuspended in 0.5 mL of PI (50 mg/L) and 1.5% of RNase A (7 mg/mL) for 30 min at 37 °C in the dark, and analyzed using flow cytometry analysis (BD FACS Calibur; BD Biosciences). The cells were classified as late- or early-stage apoptotic cells by staining with annexin V-FITC and PI (FITC Annexin V apoptosis detection kit-1; BD Pharmingen, San Jose, CA, USA). Briefly, the cells were collected by centrifugation at a concentration of 3 × 10^5^ cells/tube, washed twice with cold-PBS and once with 1 mL of binding buffer, and stained with 150 µL binding buffer containing 2.5 µL of annexin V-FITC and 0.1 µL of PI at room temperature for 15 min in the dark. The stained cells were subjected to flow cytometry analysis (BD FACS Calibur; BD Biosciences).

## Supplementary information

dataset1
