# Supplementary material for: Microgravity inhibits decidualization via decreasing Akt activity and FOXO3a expression in human endometrial stromal cells
Source: Sci Rep. 2019 Aug 20;9:12094. doi: 10.1038/s41598-019-48580-9 (PMC6702225; doi:10.1038/s41598-019-48580-9)
Supplement: Supplementary file 1 — dataset1 [file 41598_2019_48580_MOESM1_ESM.docx]

**Microgravity inhibits decidualization via decreasing Akt activity and FOXO3a expression in human endometrial stromal cells**

Hye-Jeong Cho^1, 2, 3^, Mi-Ock Baek^1, 2, 3,^ Sana Abdul Khaliq^1, 2, 3^, Seung Joo Chon ^4^, Kuk Hui Son^5^, Sung Ho Lee^6^, and Mee-Sup Yoon^1, 2, 3*^

^1^Department of Molecular Medicine, School of Medicine, ^2^Lee Gil Ya Cancer and Diabetes Institute, ^3^Department of Health Sciences and Technology, GAIHST, Gachon University, Incheon 21999, Republic of Korea, ^4^Department of Obstetrics and Gynecology, ^5^Department of Thoracic and Cardiovascular Surgery, Gachon University Gil Medical Center, College of Medicine, Gachon University, Incheon 21565, Republic of Korea, ^5^Department of Thoracic and Cardiovascular Surgery, Korea University, Seoul, 02841, Republic of Korea

Short title: Inhibition of decidualization via Akt/FOXO3a under microgravity

*Corresponding author:

Professor Mee-Sup Yoon, PhD.

Department of Molecular Medicine, School of Medicine, Lee Gil Ya Cancer and Diabetes Institute, Department of Health Sciences and Technology, GAIHST, Gachon University, Incheon 21999, Republic of Korea.

E-mail: [msyoon@gachon.ac.kr](mailto:msyoon@gachon.ac.kr)

Tel: 82-32-899-6067

Fax: 82-32-899-6039

**Supplementary Fig 1 (related to Fig 4). β-catenin phosphorylation was not regulated by Akt in human eSCs**

Human eSCs were serum-starved overnight, and treated with 100 nM insulin for 10 min. Cell lysate were analyzed by western blotting. All blots shown are representative of 3 to 5 independent experiments.

**Supplementary Fig 2 (related to Fig 5). The expression level of p62 decreased under simulated microgravity.**

(A-C) Human eSCs were exposed to either terrestrial gravity or SM for 36 h, lysed and subjected to qRT-PCR (A) and western blotting (B). (C) Western blot images were analyzed using ImageJ to determine the expression of p62 relative to tubulin. All blots shown are representative of 3 to 5 independent experiments.

**Supplementary Fig 3 (related to Fig 5). Inhibition of Akt did not affect FOXO3a expression in human eSCs**

Human eSCs were incubated with or without 1 μM Akti for the indicated times, lysed, and subjected to western blotting. All blots shown are representative of 3 to 5 independent experiments.
